# Association of runoff risk with private well contamination in a fractured dolostone aquifer

**DOI:** 10.1002/jeq2.70296

**Published:** 2026-10-29

**Authors:** Joe Heffron, Joel P. Stokdyk, Tucker R. Burch, Rachel M. Cook, Aaron D. Firnstahl, Eric O. Young, Jessica F. Sherman, Dustin Goering, Burney A. Kieke, Mark A. Borchardt

**Affiliations:** ^1^ USDA‐ARS Environmentally Integrated Dairy Management Research Unit Marshfield Wisconsin USA; ^2^ U. S. Geological Survey and USDA‐ARS Laboratory for Infectious Disease and the Environment Marshfield Wisconsin USA; ^3^ U.S. Geological Survey Upper Midwest Water Science Center Marshfield Wisconsin USA; ^4^ U.S. National Weather Service North Central River Forecast Center Chanhassen Minnesota USA; ^5^ Center for Clinical Epidemiology and Population Health Marshfield Clinic Research Institute Marshfield Wisconsin USA

## Abstract

In the United States, private well owners are responsible for monitoring and maintaining their drinking water but have little control over neighboring contamination sources and limited information about daily water quality. Potential for rapid contamination in karst settings exacerbates the challenge. The Runoff Risk Advisory Forecast (RRAF) is a real‐time online tool available in five US states that helps nutrient applicators avoid times and locations of high runoff risk. We wanted to determine if RRAF could be repurposed in areas susceptible to groundwater contamination to inform well owners of conditions when contamination is likely. Private wells were repeatedly sampled on seven dates (32–36 wells per date) and analyzed for nitrate‐nitrogen, total coliforms, and bovine manure markers. Three‐day windows of RRAF risk prior to sampling were selected post hoc, ranging from 1 to 3 days prior for manure markers to 10–12 days prior for nitrate. Contaminants were associated with RRAF values of ≥75% within these windows based on observed weather. A hydrologic association between RRAF risk and groundwater depth was also apparent. Overall, results suggest the RRAF model anticipates conditions of elevated contamination risk for susceptible wells. For nitrate, between‐well variation and delayed peak concentration suggested that well location and construction factors may be more informative than runoff events. Microbial contamination was rapid and distributed among wells, with one or more detections of coliforms in 31% of wells and bovine markers in 61% of wells. Thus, a warning system based on the RRAF model could help identify conditions of acute pathogen exposure.

## Plain Language Summary

In the United States, private wells are not regulated at the federal level. Individual well owners must monitor water quality and mitigate hazards on their own initiative, but they have little control over most factors affecting their well's water quality and few tools to assess health risks. This research aimed to evaluate an online tool for predicting surface water runoff as an early warning system for private well contamination in a region of intensive agriculture and rapid infiltration to groundwater. Runoff risk corresponded to changes in groundwater levels, indicating relevance for well water. High runoff risk was associated with microbial and nutrient contamination in 36 private wells that draw water from a vulnerable karst aquifer. This study demonstrates the feasibility of repurposing an existing tool in five US states to alert well owners of impending, acute contamination events.

**Abbreviations**
MST: microbial source tracking
RRAF: Runoff Risk Advisory Forecast

## 1 INTRODUCTION

Private wells serve 23 million US households (Murray et al., 2021), providing a crucial source of drinking water in rural communities. Factors leading to contamination of private wells can be grouped broadly into categories of source, transport, hydrogeology, and well construction. Chemical fertilizers and fecal sources, like livestock manure or residential septic effluent, are sources of nitrate or enteric pathogens (Lee & Murphy, 2020), and groundwater quality has been linked to various measures of these sources, like agricultural inputs, proximity to manure storage facilities, area of agricultural fields, and septic system density (Borchardt, Stokdyk, et al., 2021; Hamlin et al., 2022; Ransom et al., 2022; Stokdyk et al., 2025). Opportunity for transport is provided by precipitation in the form of rain and melting snow (Allen et al., 2017; Hamlin et al., 2022; Hynds et al., 2012; Murphy et al., 2020), and well construction and geologic factors can either attenuate or exacerbate rates of contamination (Allen et al., 2017; Borchardt, Stokdyk, et al., 2021; Hynds et al., 2012; Ransom et al., 2022; Stokdyk et al., 2025). Recognizing factors that contribute to groundwater contamination helps identify conditions and settings prone to water quality degradation; however, for an individual private well user, the presence and concentration of specific contaminants may often be unknown because monitoring and treatment of private well water are neither required nor common (Malecki et al., 2017).

Groundwater in karst settings is particularly vulnerable to contamination (DeSimone et al., 2009; Diston et al., 2015; Worthington & Smart, 2017). In Northeast Wisconsin, the karstic Silurian dolostone aquifer is an important source of private well water, and contamination by nitrate, wastewater, manure, and pathogens has been documented (Borchardt et al., 2011; Borchardt, Stokdyk, et al., 2021; Erb et al., 2015; Muldoon & Bradbury, 2010). This regionally important aquifer features dense, ubiquitous fracture networks allowing rapid groundwater flow, with rates of meters to tens of meters per day (Bradbury & Muldoon, 1992; Muldoon et al., 2001; Muldoon & Bradbury, 2010), and vertical fractures allow rain and snowmelt to infiltrate easily, carrying contaminants from the soil surface to the water table (Muldoon & Bradbury, 2010).

The Runoff Risk Advisory Forecast (RRAF, v. 2.1; https://runoffriskadvisory.wi.gov/) is a publicly available tool designed to inform the timing of nutrient application by predicting the risk of transporting land‐applied nutrients to nearby waterbodies. In Wisconsin, where manure from dairy farms is a prominent source of nutrients and fecal organisms on the landscape, RRAF is primarily intended to schedule manure application (Wisconsin Manure Management Advisory System, 2025). The model is operated by the National Oceanic and Atmospheric Administration's (NOAA) National Weather Service North Central River Forecast Center (NCRFC) and active in Wisconsin and other Great Lakes states (Minnesota, Michigan, Ohio, and New York; National Oceanic and Atmospheric Administration [NOAA], 2019). RRAF addresses the influence of transport factors, such as precipitation, snowmelt, soil moisture, and temperature (Wisconsin Manure Management Advisory System, 2025), on surface water contamination by manure and other fertilizers. Many of these factors can also affect transport of pathogens and other microbes to groundwater, especially in settings where rapid vertical infiltration readily connects the surface to groundwater.

This study assessed the feasibility of using RRAF to predict private well contamination in the karst aquifer of Northeast Wisconsin. Groundwater recharge has been associated with private well contamination in the region (Borchardt, Stokdyk, et al., 2021), and an initial evaluation suggested an association between RRAF v.1 and private well contamination by bovine enteric microorganisms (Borchardt et al., 2019). Study objectives were to (1) synoptically sample and analyze a cohort of 30 wells over 1 year for nitrate, fecal indicator bacteria, and bovine microbial source tracking (MST) markers; (2) evaluate spatial and temporal variability of rural well contamination within the cohort of study wells; and (3) determine whether runoff conditions modeled by RRAF are associated with groundwater contamination. RRAF's advantage in the current study is that it is free and readily available in the state. If runoff conditions predicted by RRAF are associated with contamination of private wells, it may be possible to alert well users to conditions when degraded groundwater quality is more likely. Sampling took place over 13 months and was coordinated with RRAF predictions across a wide range of runoff conditions from periods of low to high risk.

## 2 MATERIALS AND METHODS

### 2.1 Study location

Kewaunee County in Northeast Wisconsin, has a population of 20,800 (Bay Lake Regional Planning Commission, 2021). The climate is continental and influenced by proximity to Lake Michigan. Of the total land area (89,000 ha), agricultural land accounts for 68,400 ha (77%) owned by 605 farms, 42 of which house ≥500 cows (USDA NASS, 2024). The total number of private wells in the county is ∼4900 based on septic system counts and county tax parcel data (Borchardt et al., 2019). Soils in the study area range from shallow (Casco series, fine‐loamy over sandy or sandy‐skeletal, mixed, superactive, mesic Inceptic Hapludalfs) to very deep (e.g., Hortonville and Waymor, fine‐loamy, mixed, active, mesic Haplic Glossudalfs, Symco, fine‐loamy, mixed, active, mesic Aquollic Hapludalfs, and Omro, clayey over loamy, mixed, active, mesic Typic Hapludalf, series) and are predominately calcareous, loamy, and well‐drained to excessively drained (e.g., Hortonville, Casco, Omro, Waymor series) (NRCS, 2022). Most of Kewaunee County's pumped groundwater is held in a Silurian dolostone aquifer. The densely fractured dolostone and thin soils contribute to rapid groundwater recharge and high influence of surface water on groundwater (Muldoon & Bradbury, 2010). Groundwater quality in Kewaunee and its neighboring counties has been studied extensively, showing evidence of contamination by coliform bacteria, enteric pathogens, and nitrate (Borchardt et al., 2019; Borchardt, Stokdyk, et al., 2021; Erb et al., 2015).

### 2.2 Private well selection and experimental design

Participation letters were sent to 200 private well owners selected randomly from 621 wells sampled previously (Borchardt, Stokdyk, et al., 2021). Of the 89 respondents (45% response rate), 36 well owners were randomly selected for the study, with a goal of ≥30 wells sampled in each sampling event. Wells were selected irrespective of previous contamination, well characteristics, and surrounding land use, which was characterized for the area near wells as described by Borchardt, Stokdyk, et al. (2021). Cultivated fields were within 229 m of 32 of the 36 wells and comprised 0%–96% of the nearby land area (0–15.9 ha within 229 m); bedrock depth ranged <1–55 m (median, 10.7 m; Table 1).

**TABLE 1:** Characteristics of the private wells (*n* = 36) in this study (Borchardt, Stokdyk, et al., 2021).

Characteristic	Unit	Median (minimum, maximum)	Data not available
Cultivated fields within 229 m	ha	6.93 (0, 15.9)	0 wells
Distance to nearest manure storage	m	970 (271, 2137)	0 wells
Bedrock depth	m	10.7 (0.91, 55.2)	0 wells
Casing depth	m	18.9 (12.2, 64)	10 wells
Elevation	m	227 (186, 257)	0 wells
Groundwater depth at construction	m	15.2 (1.22, 31.1)	9 wells

Wells were sampled synoptically on seven dates from November 2022 to December 2023, with 32–36 samples per event. Sampling dates were selected to capture a range of hydrologic conditions. In addition, two sampling dates (April 19 and April 26, 2023) were clustered shortly after the County's annual winter manure spreading ban was lifted (January 1–April 15). The final dataset included 235 samples from 36 wells (1–7 samples/well).

Samples were analyzed for nitrate, total coliforms, and bovine MST markers. Bovine MST was included because dairy manure is a major fecal input in the study area and RRAF is intended to inform manure management in Wisconsin. Bovine MST markers were previously associated with groundwater recharge in the study area and detected in more wells than human MST markers (Borchardt, Stokdyk, et al., 2021).

### 2.3 Sample collection

For each event, all samples were collected on a single day. Samples were collected by sterilizing the tap with a flame or alcohol, flushing and discarding at least 2 L of water, then collecting two 1 L samples and one 500 mL sample in presterilized polypropylene bottles. Samples were kept on ice, transported to the lab, and stored at 4°C overnight.

Samples were processed within 24 h of collection. One liter samples were spiked with 1 mL of *Moraxella bovis* (positive recovery control; Schering–Plough Piliguard Pinkeye‐1 Trivalent) and 10 mL of 2.5 M MgCl_2_, then filtered through two separate 0.45‐µm MCE membrane filters (Millipore Sigma), resulting in each sample having duplicate A and B filters. Using a 6‐port manifold (Millipore Sigma) and new sterilized glassware for each sample, six A filters were processed at a time, followed by the same six samples passed through B filters. All filters were transferred with flame‐sterilized tweezers to 50‐mL conical tubes and frozen at −80°C.

### 2.4 Sample analysis

Each 500 mL sample was aliquoted for total coliform and nitrate analysis. A 100 mL subsample was used for total coliform detection with the IDEXX Colilert kit, according to manufacturer instructions as detailed in Section S1.1. An 8 mL subsample of the homogenized remaining volume was analyzed for nitrate‐nitrogen (Method 4500‐NO_3_ F; [APHA, 1995]) on a QuikChem 8500 (Lachat Instruments) using method 10‐107‐04‐1‐C. Samples over the calibration curve were diluted with reverse osmosis water. Samples below the detection limit (0.002 mg L^−1^ NO_3_‐N) were assigned a value of 0.001 mg L^−1^ for statistical analysis. Nitrate analysis was included in the study after the first sampling date (November 16, 2022).

Frozen MCE filters were thawed and extracted using the Qiagen QIAamp DNA Mini Kit and the QIAcube Connect (Qiagen), as detailed in Section S1.2. Extracted samples were analyzed by quantitative polymerase chain reaction (qPCR) for five bovine MST markers: bovine adenovirus (serotypes 4–8; hexon), bovine polyomavirus (*VP1*), Bacteroidales‐like CowM2 (DHIG domain protein), Bacteroidales‐like CowM3 (HD super family hydrolase), and ruminant *Bacteroides* (16S rRNA). Primer and probe sets are listed in Table S1. The qPCR was performed with a LightCycler 480 and LightCycler 480 Probes Master kit (Roche Diagnostics). Detailed qPCR parameters are provided in Section S1.2; standard curve parameters and 95% limits of detection (LODs) are reported in Table S2.

Each filter was extracted and analyzed in duplicate, producing four qPCR replicates per target per sample. Nucleic acid extraction failed and could not be repeated for B filters from a subset of samples collected on February 8, 2023 (16 samples, 0 positive), and April 19, 2023 (10 samples, 1 positive). Analytical sensitivity for these samples was lower than for other wells and dates. Detections are reported at the sample‐level for descriptive purposes, but detections were represented at the filter level for modeling purposes to account for this difference in sensitivity (Section 2.6). Quality control measures and the Environmental Microbiology Minimum Information (EMMI) checklist (Borchardt, Boehm, et al., 2021) for this study are provided in Section S1.3 (Figure S1).

### 2.5 Data sources

Surface runoff risk was characterized using the Runoff Risk model, v. 2.1 (NOAA, 2019). The model incorporates hourly observed and forecast precipitation and temperature data into the gridded Sacramento Soil Moisture Accounting and Snow‐17 models to simulate continuous soil moisture, snowpack, and runoff. In the Wisconsin implementation, runoff risk is updated four times daily to incorporate observed weather into the risk calculation (Wisconsin Manure Management Advisory System, 2025). The severity of predicted runoff risk, expressed as a percentage, is relative to historic runoff volumes in each 2 km × 2 km geographic cell (NOAA, 2019). Participating wells were matched to the containing model cell based on latitude and longitude. We used post hoc model calculations based on observed daily weather conditions, rather than forecasted weather conditions as used in the RRAF online tool. These post hoc weather inputs allowed us to test the association between modeled runoff risk and contamination without the uncertainty of weather predictions. Daily runoff risk percentile values were used without categorizing by risk level or frozen conditions.

To test the relationship between surface runoff and groundwater recharge, runoff risk percentiles were compared to groundwater levels from a US Geological Survey monitoring well in Kewaunee County (KW‐25/24E/34‐0183) for the study period. Daily maximum groundwater depth (feet below land surface) was sourced from the US Geological Survey National Water Information System (USGS, 2025).

### 2.6 Statistical analysis

Univariate relationships between runoff risk percent and three outcome variables (detection of any bovine MST markers, detection of total coliforms, and nitrate‐N concentrations) were characterized by examining the association between the outcome variable on a given sampling date (the anchor date) and the maximum runoff risk percent in a time window prior to this date. The analytic framework for all outcomes was similar, although there were differences for detection outcomes (total coliforms and bovine MST markers) versus continuous‐scaled outcomes (i.e., nitrate concentration).

We evaluated maximum runoff risk percent in 3‐day windows from days one through 21 prior to the anchor date for all contaminants (i.e., 1–3, 2–4, 3–5, …, 19–21). A length of 3 days was chosen for consistency with the prediction window length of the Wisconsin RRAF online tool and to balance stability and timeliness (more stable estimates than shorter window lengths and more timely estimates than longer window lengths). We selected a single 3‐day window for each outcome. For detection outcomes, time windows were screened using risk as categorized by the RRAF tool (Wisconsin Manure Management Advisory System, 2025): No runoff expected (0% to <30% risk), low risk (30% to <60%), moderate risk (60% to <90%), and severe risk (≥90%). The windows were assessed based on (1) *p*‐values for two‐sided Fisher's exact tests from crosstabulations of contaminant detection status versus runoff risk category, and (2) examination of observed patterns in the ratio of detection rates in elevated risk categories relative to the “no runoff expected” category. For the nitrate concentration outcome, time windows were evaluated based on goodness‐of‐fit statistics from gamma regression models and relevant graphics (Garson, 2013; Pan, 2001). The chosen time windows should be interpreted as approximate in contrast to fixed/universal transport rules. Additional details regarding time window selection are provided in Section S2 and Table S3. For the selected 3‐day windows, we computed model‐based estimates of the ratio of microbe detection rates (or mean nitrate concentrations) for each observed maximum runoff risk percent value >0 (in 5% increments) versus when maximum runoff risk percent = 0 (i.e., rate for maximum runoff risk = 5% vs. rate for maximum runoff risk = 0%, 10% vs. 0%, 15% vs. 0%, etc.). A ratio was considered significantly elevated when its lower 95% pointwise confidence limit was greater than the null value of 1.0. For detection outcomes, the ratios were estimated using log‐binomial regression models (McNutt, 2003).

Maximum runoff risk percent was represented as a natural cubic spline in all regression models (Hastie et al., 2001). Wells were sampled on multiple dates, and each sampling date included two filters for bovine MST markers. Therefore, in models, data for nitrate concentration and total coliforms were characterized as having sampling dates nested within wells, whereas the bovine MST marker outcome had filter‐level nucleic acid extractions nested within wells and sampling dates to account for uneven analytical replicates. Generalized estimating equations were used to account for non‐independence of the source data (Liang & Zeger, 1986). In nitrate concentration analyses, values of 0.001 mg NO_3_‐N L^−1^ (LOD/2) were imputed for non‐detects. Analyses were conducted using SAS 9.4 (SAS Institute, Inc.).

## 3 RESULTS

### 3.1 Occurrence of contamination

Coliform bacteria, bovine fecal markers, and/or elevated nitrate concentrations were detected on all sampling dates and in 29 of 36 study wells (81%) (Figure 1). Seventeen of 202 total samples (8.4%) exceeded the maximum contaminant level (MCL) for nitrate (10 mg NO_3_‐N L^−1^), and 69 (34%) exceeded the national background nitrate concentration (1 mg NO_3_‐N L^−1^), suggesting anthropogenic influence on groundwater quality (Dubrovsky et al., 2010). By contrast, 100 samples (50%) were below the LOD of 0.002 mg NO_3_‐N L^−1^. Coliform bacteria were detected in 11% of samples (27/235) and bovine fecal markers in 13% of samples (31/235). Coliforms and manure markers co‐occurred in only four samples. At the well level, 13 of 36 wells (36%) had nitrate levels indicating anthropogenic influence at least once in the study, and five had nitrate exceeding the MCL. Eleven of 36 wells (31%) had a total coliform detection. In contrast, 22 wells had at least one bovine MST detection (61%). Detailed tabulation of outcomes by date and well is provided in Tables S4 and S5, respectively; the dataset for this study can be found in the National Agricultural Library AgData Commons (https://doi.org/10.15482/USDA.ADC/31114333.v2).

**FIGURE 1:**
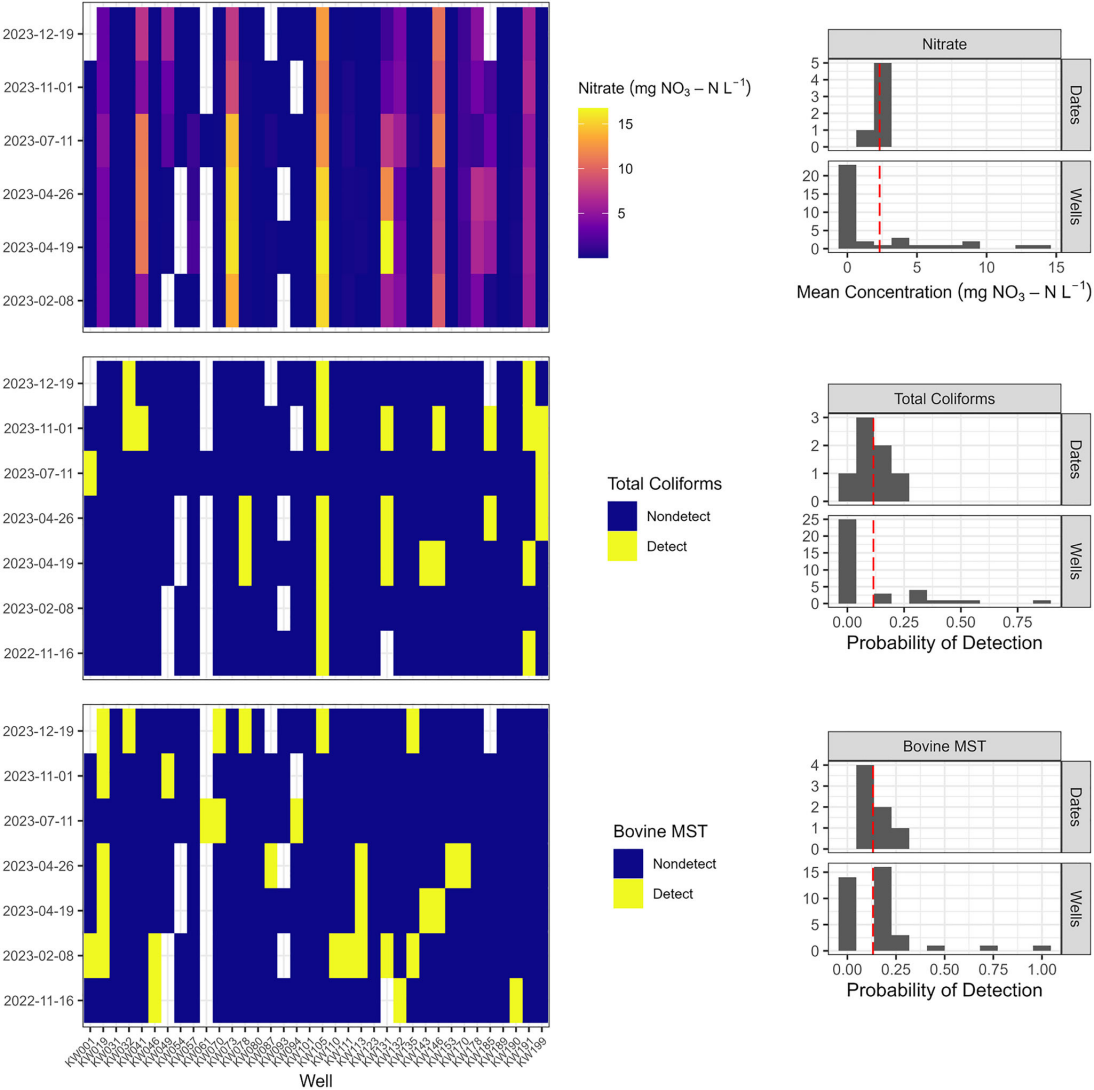
Concentration of nitrate (mg NO_3_‐N L^−1^) and detection of total coliforms (TC) and microbial source tracking (MST) markers for individual wells over the duration of the study. Blank (white) spaces in the heatmaps indicate the sample was not collected or did not pass quality controls. Histograms show contamination outcomes across wells on a given sampling date (“Dates”) versus within individual wells over time (“Wells”). Dashed vertical lines (in red) represent the overall mean concentration (nitrate) or probability of detection (TC and bovine MST).

Water quality varied between wells but comparatively less between sampling dates (Figure 1). The mean nitrate concentration for all wells by date varied by only 1.05 mg NO_3_‐N L^−1^ (min: 1.82 mg NO_3_‐N L^−1^, max: 2.87 mg NO_3_‐N L^−1^; Table S4), while mean well‐level nitrate concentration ranged from <0.002 to 14.0 (Table S5). Of the 13 wells with nitrate levels indicating anthropogenic influence, 10 exceeded 1 mg NO_3_‐N L^−1^ in all six samples (Table S5). Similarly, 25 wells were never positive for total coliforms, while eight of 11 coliform‐positive wells were positive multiple times. Bovine fecal markers were more evenly distributed among wells, as 17 of 22 MST‐positive wells were positive only once (Table S5).

### 3.2 Surface runoff as an indicator of groundwater recharge

Our investigation of runoff risk as a potential indicator of groundwater contamination hypothesizes that surface and vadose zone contaminants are transported to groundwater primarily through heavy precipitation/snowmelt events. To justify this mechanism of transport, we compared daily runoff risk percentiles from the RRAF model to groundwater levels (USGS, 2025). Groundwater levels exhibited the typical pattern of significant recharge in early spring with gradually declining levels through summer. High runoff risk percentiles corresponded to decreasing depth to groundwater (recharge), whereas periods of no runoff risk (<25%) corresponded to increasing depth to groundwater (Figure 2). Groundwater recharge was roughly concurrent with high runoff risk events, and daily change in groundwater depth was significantly correlated to runoff risk for the previous day (Pearson: −0.53, *p* < 2.2 × 10^−16^; Figure 2, inset). This correlation suggests a rapid hydrological response to surface runoff and a potential mechanism for rapid intrusion of surface contaminants into the area's unconfined, karstic aquifer during runoff‐producing events.

**FIGURE 2:**
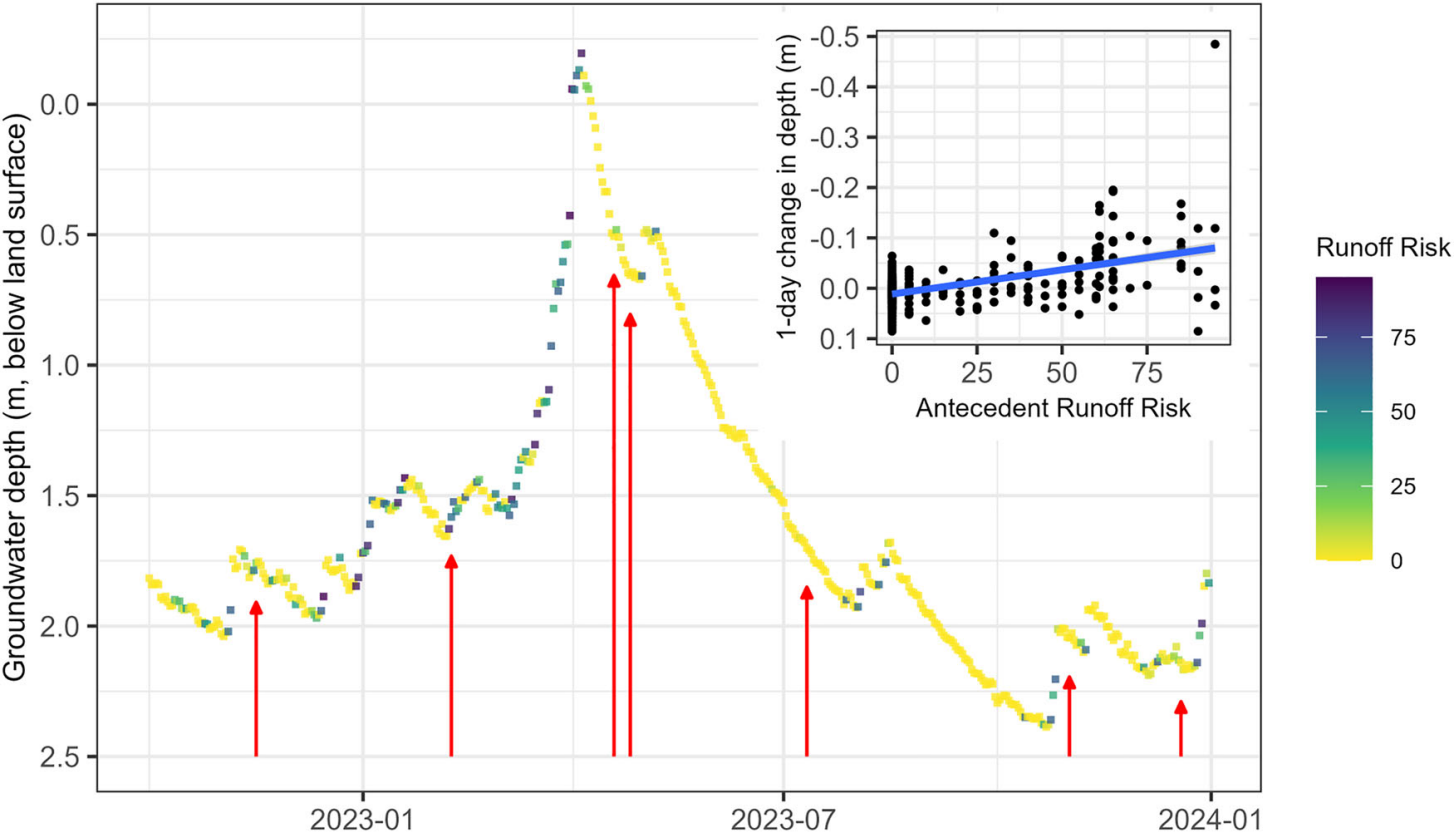
Comparison of runoff risk and depth to water level for USGS monitoring well KW‐25/24E/34‐0183. Vertical arrows (in red) indicate sampling dates. Inset: Day‐to‐day change in groundwater depth as a function of the antecedent (prior day's) runoff risk.

### 3.3 Association of contamination with surface runoff model

Detection of bovine MST markers, coliform bacteria, and nitrate concentrations in private wells was associated with runoff risk prior to sample collection. The selected time windows associated with contamination were 1–3 days prior to sampling for bovine MST markers, 5–7 days for total coliforms, and 10–12 days for nitrate concentration (see Section S2 for additional details). Compared to contamination estimates for runoff risk percent = 0, estimates for values ≥75 were significantly elevated for all outcomes. The estimated ratios for maximum runoff risk percent values ≥75 were 1.29–1.46 (corresponding to mean concentrations of 2.198–2.490 vs. 1.709) for nitrate concentration (Figure 3A), 2.21–4.80 (corresponding to detection rates of 0.179–0.389 vs. 0.081) for total coliforms detection (Figure 3B), and 2.32–3.11 (corresponding to detection rates of 0.148–0.198 vs. 0.064) for bovine MST marker detection (Figure 3C).

**FIGURE 3:**
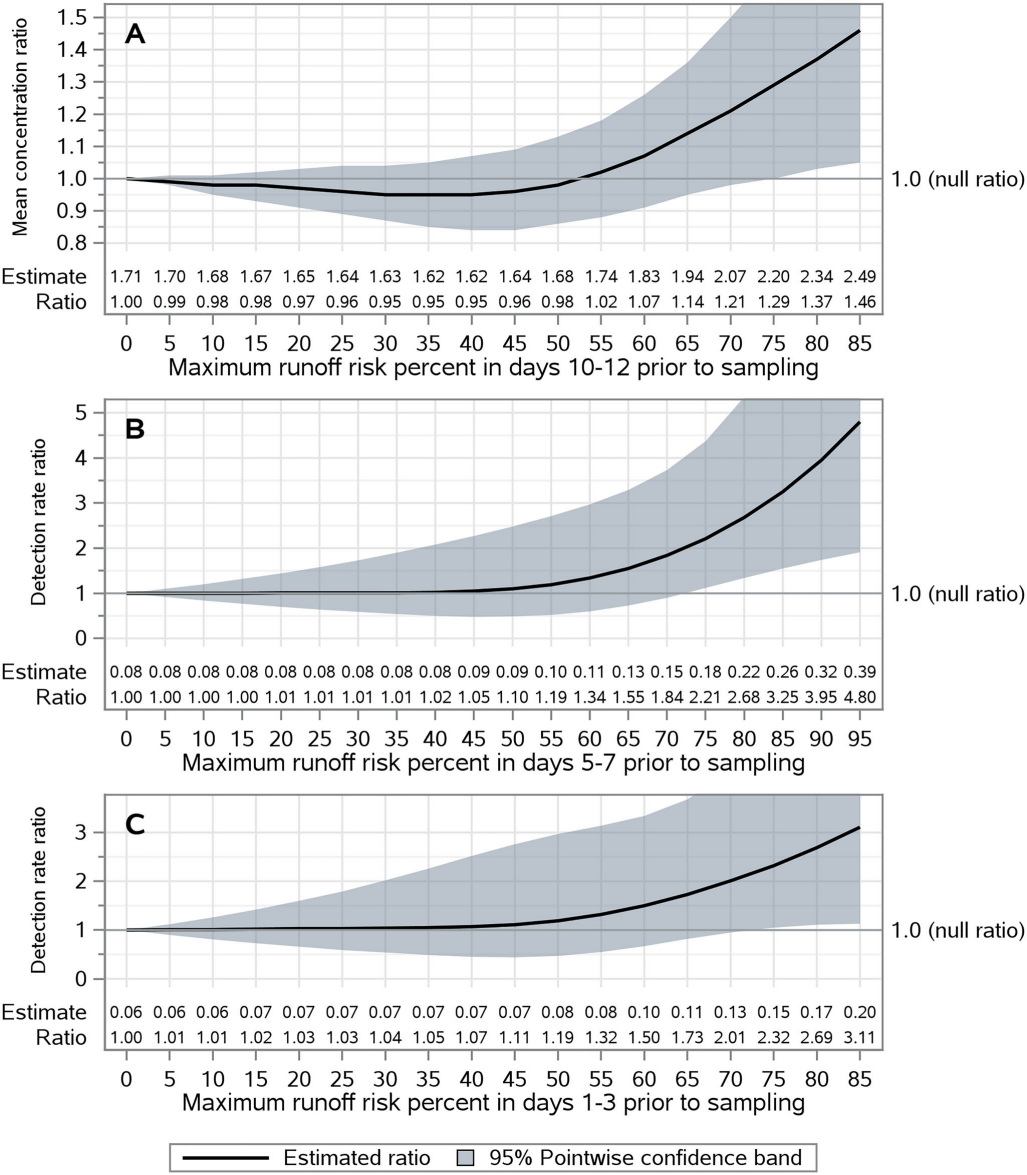
Ratios by runoff risk for (A) mean nitrate concentration, (B) total coliform (TC) detection, and (C) bovine microbial source tracking (MST) detections. Denominator values for estimated ratios are those for maximum runoff risk percent = 0 (i.e., 1.71 [A], 0.08 [B], and 0.06 [C]). Statistical significance at the 0.05 level is indicated when the lower confidence limit is >1.0.

## 4 DISCUSSION

### 4.1 Surface runoff as a risk factor for groundwater contamination

This study sought to associate the potential for surface runoff as computed by RRAF forecasts with contamination of private wells in a karst region of Northeast Wisconsin. RRAF is a tool available in five US states and is intended to protect surface waters from contamination by advising farmers when there is an elevated risk of runoff. The local hydrogeology promotes rapid infiltration and subsurface transport of contaminants, on the order of 2–7 days for microbial fecal markers (Borchardt, Stokdyk, et al., 2021). Thus, the surface conditions represented in RRAF forecasts may be informative of private well contamination in this setting. Using RRAF to predict groundwater contamination expands the tool's applicability. With further validation, homeowners may be able to use RRAF to understand when their well water is at risk of contamination, for example, by signing up for SMS or email alerts offered in some states (Michigan State University, 2026; Minnesota Department of Agriculture, 2026). RRAF thereby provides a preexisting, cost‐effective, and accessible tool for private well owners to anticipate bouts of potentially poor water quality.

We sampled a cohort of private wells repeatedly over time and across a range of RRAF forecast values, and coliforms, bovine‐specific MST markers, and nitrate demonstrated positive associations with RRAF forecasts (Figure 3). Thus, groundwater contamination in this setting was associated with surface runoff conditions even without incorporating well‐specific geology and construction factors, though the inclusion of local factors would be necessary to distinguish the susceptibility of individual wells within event‐driven risk. Some well contamination also occurred in conditions of little or no expected runoff, but contamination was more likely when runoff risk was high. While current County recommendations advise against spreading manure in “severe” RRAF conditions (Kewaunee County, WI, 2025; Wisconsin Manure Management Advisory System, 2025), results suggest even “moderate” runoff risk (categorized as 60 to <90 percentiles) may lead to elevated groundwater contamination. Estimated rates of coliform and bovine fecal marker detection at the 75th percentile were more than double those at zero risk, while nitrate concentrations were 29% higher than at zero risk (Figure 3).

Furthermore, groundwater contamination in this study was associated with RRAF model outcomes without detailed information on contaminant sources. Our target contaminant source was dairy manure, which is unevenly distributed geographically and temporally. For example, agricultural intensity varied among well locations (Table 1), and we expect more manure application in spring and late fall when fields are bare of crops. Despite uncertainty around contaminant sources, the strong association between RRAF values and groundwater depth (Figure 2) demonstrates that RRAF predictions may inform conditions for transport of contaminants to groundwater.

While groundwater levels at the monitoring well responded rapidly to runoff risk events, infiltration of water from the surface and vadose zone typically lags behind hydraulic head (Hamilton, 2012). Based on nitrate concentrations and microbial detections, we selected 3‐day windows of relevant runoff risk to account for delay between runoff events and groundwater infiltration. Contamination outcomes often did not coincide (Figure 1), and the selected window for contamination after a runoff event varied by contaminant. Bovine fecal markers seemed to appear more quickly following days with high runoff risk (1–3 days), followed by coliforms (5–7 days), and finally elevated nitrate concentrations (10–12 days; Section S2). These approximate windows for contaminant transport accord well with previous reports. Similarly short transport times for microbes have been previously observed in karstic dolostone aquifers. A previous study in the region also found evidence for rapid transport (2–7 days) of microbial contaminants following precipitation and groundwater recharge (Borchardt, Stokdyk, et al., 2021). In a similar hydrogeological setting, Arnaud et al. (2015) detected elevated *Escherichia coli* concentrations within 7 days of liquid swine manure application. Such rapid microbial transport is likely due to preferential flow (Arnaud et al., 2015; Voisin et al., 2018). Sinkholes, vertical fissures, and bedrock features contributing to preferential flow have been well documented in our study area (Erb et al., 2015; Luczaj, 2013).

The selected window for nitrate was slightly delayed compared to microbial contaminants, consistent with expected differences in contaminant transport (Hunt & Johnson, 2017). In contrast to microbial detections, nitrate contamination was assessed as a continuous outcome (concentration); while early detection is favored by preferential flow, changes in concentration depend on bulk water transport through a mix of fast and slow pathways (Kuehner et al., 2025). Preferential flow paths form the leading edge of a contaminant plume but do not typically represent the majority of bulk water transport (Hunt & Johnson, 2017). In a study of private wells in the same aquifer as the current study, influent water from snowmelt and precipitation events was detectable as deflections of isotopes ^2^H and ^18^O within 7 days but lasted on the order of days to weeks (Luczaj et al., 2023). The Day 10–12 window for nitrate contamination used in the current study aligns with this broad timeline, consistent with bulk water transport. Solutes like nitrogen are also likely to diffuse into soil pore spaces, extending the plume duration, while colloids like bacteria tend to travel faster because fluid drag directs colloids toward the center of advective flow (Hunt & Johnson, 2017).

In addition, the unsaturated zone may act as a sink and source for bacteria and nitrate, both delaying and extending contamination plumes. Ammonia nitrogen from manure and fertilizer can be converted to organic nitrogen in the root zone, only to leach nitrate gradually over time. The biogeochemical time lag of soil organic nitrogen can be on the order of decades (Van Meter et al., 2016). Even in the absence of new nitrogen inputs, soil nitrogen reserves can continue to contaminate groundwater for years (Hamilton, 2012; Kuehner et al., 2025; Van Meter et al., 2016). Root and vadose zones can also serve as a sink for bacteria from the surface and gradual source of environmental bacteria seeding to groundwater over shorter time scales (Arnaud et al., 2015; Voisin et al., 2018). Accordingly, contamination plumes for nitrate and coliforms are likely to continue beyond the windows identified in this study.

### 4.2 Rates of contamination in context

Wells were sampled repeatedly for the current study, increasing the likelihood of detecting contamination at the well level. At the sample level, rates of nitrate exceedance (NO_3_‐N > 10 mg L^−1^) found here (8.4%) were comparable to rates for private wells in Wisconsin for the same year (7.3%; DATCP, 2024) and lower than the roughly one third of 47 private wells that exceeded the MCL in agricultural areas of the neighboring state of Iowa (Bradley et al., 2023). The sample‐level rate of coliforms contamination in the current study (11%) was less than for rural wells in Wisconsin (18%) (Knobeloch et al., 2013) and Iowa (28%) (Bradley et al., 2023) as well as for wells north of the current study area in Ontario, Canada (20%–30%) (Petculescu et al., 2022).

For Kewaunee County, rates of nitrate exceedance in the current study were similar to a county‐wide, random sampling of private wells in 2015–2016 (7%) and lower than rates of 10%–14% reported for samples collected voluntarily in 2023 (Borchardt, Stokdyk, et al., 2021; Masarik, 2023). Sample‐level coliforms contamination was lower than rates previously reported for private wells in Kewaunee County (15%–23%) (Masarik, 2023), and the sample‐level (13%) and well‐level (61%) contamination rates for bovine/ruminant MST markers bracketed results from the previous study (34%) from which our study wells were randomly sampled (Borchardt, Stokdyk, et al., 2021).

Over the past decade, local and state lawmakers enacted standards for manure storage and application in areas with less than 6.1‐m depth to Silurian bedrock (Kewaunee County Ordinances, 2016; Wisconsin Administrative Code, 2018). The Kewaunee County Land and Water Conservation Board refers to the Wisconsin RRAF tool as guidance for complying with manure application restrictions, admonishing, “When it is RED, DO NOT spread!” (Kewaunee County, WI, 2025), where red coloration indicates “severe” forecasts (≥90 risk percentiles; Wisconsin Manure Management Advisory System, 2025). To the extent this guidance was followed, manure would not have been applied during periods of ≥90% runoff risk near wells in our study with less than 6.1‐m depth to dolostone bedrock (17 of 36 wells total; Table S6).

Weather conditions may have limited transport of total coliforms and bovine fecal markers. Kewaunee County was in drought for much of the summer of 2023 (June through October), and conditions remained abnormally dry through the December sampling date (NIDIS, 2025). The study period included the full range of runoff risk severities, but prolonged drought limited variation to an extent, particularly during the fall manure spreading season in 2023. From the beginning of June to the end of the sampling period (December 19, 2023), 188 of 202 days (93%) had median RRAF percentiles among study wells in the “No runoff expected” range. Despite these limitations, modeled runoff risk was significantly associated with all three contamination outcomes: nitrate, total coliform, and bovine fecal markers.

### 4.3 Limitations

Samples were collected over 13 months and represent a range of RRAF values, but below‐average precipitation during the study may not fully represent environmental conditions in other years. Study wells included a range of geology and construction characteristics that can influence susceptibility to contamination but may not represent characteristics of all wells in the county; repeated sampling was favored over inclusion of more wells to achieve the study objective, and outcomes were based on 235 samples. Though sufficient to identify significant associations with runoff risk, the relatively low frequency of microbial detection limited statistical power and likely reduced our ability to differentiate the effects of additional risk factors. Well‐level factors may influence susceptibility, so incorporating local variables like geology, well construction, and proximity to fecal sources might improve prediction of contamination in individual wells. Nonetheless, the role of transport in this setting was evident, as no wells were continually positive for microbes and contamination was associated with runoff risk.

This study used RRAF predictions that incorporated observed weather conditions. Daily RRAF outputs based on forecasted weather identified elevated runoff risk (≥75% risk) with 70.8% recall and 98.5% specificity compared to outputs based on observed weather (Figure S2). Validation is therefore needed before using RRAF outputs based on weather forecasts as an early warning for groundwater contamination. However, the Wisconsin RRAF tool updates four times daily with observed weather conditions (Wisconsin Manure Management Advisory System, 2025). Observed data could serve as a just‐in‐time warning of likely conditions for contamination.

The windows of relevant runoff risk for each contaminant were selected in this study based on a post hoc assessment of the data. The likelihood of contamination was elevated during the selected time windows, though contamination may also occur outside these windows. These exploratory values were physically plausible and in keeping with previously reported contaminant transport times for the same region (Section 4.1), but they should not be interpreted as generalizable to other study periods or locations. In addition, the karst aquifer exhibits high susceptibility to contamination and rapid transport of contaminants (Muldoon & Bradbury, 2010). The transport times and contamination potential found for wells in this karst aquifer cannot be reliably extended to other aquifers or locations.

### 4.4 Outlook for predicting groundwater contamination from surface runoff

We found an association between contamination in private wells and prior modeled surface runoff risk in the unconfined, fractured aquifer of Northeast Wisconsin. Microbial transport was rapid (1–7 days), consistent with expectations for karst geology (Bradbury & Muldoon, 1992; Muldoon et al., 2001; Muldoon & Bradbury, 2010). The selected window of elevated nitrate occurred farther from the anchor date (10–12 days). This potential delay renders RRAF less valuable for avoiding peak nitrate contamination. Predicting acute contamination events may be less relevant for nitrate because nitrate is a persistent contaminant (Hamilton, 2012; Van Meter et al., 2016) and toxicity in drinking water is based on chronic exposure (Chambers et al., 2022; Lin et al., 2023). Decision support tools that incorporate nitrate sources and fate are available (Juckem et al., 2024), but are typically not well suited for preferential flow or karst aquifers (Juckem, U.S. Geological Survey, personal [written] communication, March 6, 2026). Considering the paucity of real‐time information available to private well owners, even an approximate window of peak contamination would be valuable for planning well testing, treatment, and/or alternative water sources.

The onset and duration of contamination following runoff events likely varies not only between contaminants, but also between wells due to local factors like distance to contamination sources, vadose zone thickness, pathways for preferential flow, and surface soil hydrology (Arnaud et al., 2015; Borchardt, Stokdyk, et al., 2021; Green et al., 2008; Voisin et al., 2018). We observed greater overall variation between wells than within each well over time, which may reflect between‐well differences in land use, hydrogeology, and well construction (Table 1) that have been associated with private well contamination in the County (Borchardt, Stokdyk, et al., 2021). For example, 12 of 13 wells with occasional to frequent nitrate concentrations indicating anthropogenic influence were sited in areas with less than 20‐m depth to bedrock (Figure S3). Borchardt, Stokdyk, et al. (2021) showed that nitrate and coliforms contamination of private wells in the county decreased with increasing depth to bedrock, with significant reductions observed where bedrock depth was ≥40 m for nitrate and 10 m for coliforms. In a study of the same region, direct recharge from snowmelt and precipitation constituted up to 13% of produced water in a private well with shallow depth to bedrock (1.2 m), but generally <2% for wells with a thicker unsaturated zone (>16 m) (Luczaj et al., 2023). Since elevated nitrate tended to be persistent in affected wells, atemporal hydrogeologic factors like depth to bedrock may be a more relevant risk factor for homeowners than surface runoff.

We do not always know when or where sources of contamination may appear on the landscape, and well owners lack control over sources that exist beyond their property. However, this study demonstrated a clear relationship between runoff risk and both groundwater recharge and likelihood of well water contamination. Therefore, RRAF indicates conditions favorable for contamination if the source is present and the well is susceptible. With further validation, RRAF could empower homeowners to schedule well testing and enact precautions. Local agencies with well testing programs might also use the RRAF tool for outreach to homes that have previously tested positive for microbial contamination.

## AUTHOR CONTRIBUTIONS

**Joe Heffron**: Data curation; formal analysis; supervision; visualization; writing—original draft; writing—review and editing. **Joel P. Stokdyk**: Conceptualization; data curation; investigation; methodology; project administration; supervision; writing—original draft; writing—review and editing. **Tucker R. Burch**: Conceptualization; funding acquisition; investigation; methodology; project administration; supervision; writing—original draft; writing—review and editing. **Rachel M. Cook**: Data curation; formal analysis; investigation; methodology; validation; writing—original draft; writing—review and editing. **Aaron D. Firnstahl**: Conceptualization; investigation; methodology; validation; writing—original draft; writing—review and editing. **Eric O. Young**: Formal analysis; methodology; supervision; writing—original draft; writing—review and editing. **Jessica F. Sherman**: Formal analysis; investigation; methodology; writing—original draft; writing—review and editing. **Dustin Goering**: Data curation; formal analysis; methodology; software; writing—review and editing. **Burney A. Kieke Jr**.: Conceptualization; formal analysis; methodology; writing—original draft; writing—review and editing. **Mark A. Borchardt**: Conceptualization; funding acquisition; methodology; writing—review and editing.

## CONFLICT OF INTEREST STATEMENT

The authors declare no conflicts of interest.

## Supporting information



Supporting Information

## Data Availability

The data generated in this study can be found in the National Agricultural Library AgData Commons. (https://doi.org/10.15482/USDA.ADC/31114333.v2).

## References

[jeq270296-bib-0001] Allen, A. S. , Borchardt, M. A. , Kieke, B. A. , Dunfield, K. E. , & Parker, B. L. (2017). Virus occurrence in private and public wells in a fractured dolostone aquifer in Canada. Hydrogeology Journal, 25(4), 1117–1136. 10.1007/s10040-017-1557-5

[jeq270296-bib-0002] APHA . (1995). Standard methods for the examination of water and wastewater (19th ed.). American Public Health Association; American Water Works Association; Water Environment Federation.

[jeq270296-bib-0003] Arnaud, E. , Best, A. , Parker, B. L. , Aravena, R. , & Dunfield, K. (2015). Transport of *Escherichia coli* through a thick vadose zone. Journal of Environmental Quality, 44(5), 1424–1434. 10.2134/jeq2015.02.0067 26436260

[jeq270296-bib-0004] Bay Lake Regional Planning Commission . (2021). Kewaunee County housing study (Final Report). Kewaunee County housing study. https://baylakerpc.org/wp‐content/uploads/2025/04/Kewaunee_County_Housing_Study_‐Final.pdf

[jeq270296-bib-0005] Borchardt, M. A. , Boehm, A. B. , Salit, M. , Spencer, S. K. , Wigginton, K. R. , & Noble, R. T. (2021). The environmental microbiology minimum information (EMMI) guidelines: QPCR and dPCR quality and reporting for environmental microbiology. Environmental Science & Technology, 55(15), 10210–10223. 10.1021/acs.est.1c01767 34286966

[jeq270296-bib-0006] Borchardt, M. A. , Bradbury, K. R. , Alexander, E. C. , Kolberg, R. J. , Alexander, S. C. , Archer, J. R. , Braatz, L. A. , Forest, B. M. , Green, J. A. , & Spencer, S. K. (2011). Norovirus outbreak caused by a new septic system in a dolomite aquifer. Groundwater, 49(1), 85–97. 10.1111/j.1745-6584.2010.00686.x 20199588

[jeq270296-bib-0007] Borchardt, M. A. , Muldoon, M. A. , Hunt, R. J. , Bonness, D. E. , Firnstahl, A. D. , Kieke, B. A. , Owens, D. W. , Spencer, S. K. , & Stokdyk, J. P. (2019). Assessing groundwater quality in Kewaunee County, Wisconsin and characterizing the timing and variability of enteric pathogen contamination within the dolomite aquifer in Northeastern Wisconsin (Final Report DNR Project Number 227). Wisconsin Geological and Natural History Survey. https://digital.library.wisc.edu/1711.dl/LDFUDOJUZZPNA8J

[jeq270296-bib-0008] Borchardt, M. A. , Stokdyk, J. P. , Kieke, B. A. , Muldoon, M. A. , Spencer, S. K. , Firnstahl, A. D. , Bonness, D. E. , Hunt, R. J. , & Burch, T. R. (2021). Sources and Risk Factors for Nitrate and Microbial Contamination of Private Household Wells in the Fractured Dolomite Aquifer of Northeastern Wisconsin. Environmental Health Perspectives, 129(6), 067004. 10.1289/EHP7813 PMC8221036 34160249

[jeq270296-bib-0009] Bradbury, K. R. , & Muldoon, M. A. (1992). Hydrogeology and groundwater monitoring of fractured dolomite in the upper Door County priority watershed, Door County, Wisconsin (Final Report). Wisconsin Geological and Natural History Survey. https://wgnhs.wisc.edu/catalog/publication/000776/resource/wofr199202

[jeq270296-bib-0010] Bradley, P. M. , Kolpin, D. W. , Thompson, D. A. , Romanok, K. M. , Smalling, K. L. , Breitmeyer, S. E. , Cardon, M. C. , Cwiertny, D. M. , Evans, N. , Field, R. W. , Focazio, M. J. , Beane Freeman, L. E. , Givens, C. E. , Gray, J. L. , Hager, G. L. , Hladik, M. L. , Hofmann, J. N. , Jones, R. R. , Kanagy, L. K. , … Ward, M. H. (2023). Juxtaposition of intensive agriculture, vulnerable aquifers, and mixed chemical/microbial exposures in private‐well tapwater in northeast Iowa. Science of the Total Environment, 868, 161672. 10.1016/j.scitotenv.2023.161672 PMC9976626 36657670

[jeq270296-bib-0011] Chambers, T. , Douwes, J. , Mannetje, A. , Woodward, A. , Baker, M. , Wilson, N. , & Hales, S. (2022). Nitrate in drinking water and cancer risk: The biological mechanism, epidemiological evidence and future research. Australian and New Zealand Journal of Public Health, 46(2), 105–108. 10.1111/1753-6405.13222 35238441

[jeq270296-bib-0012] DeSimone, L. A. , Hamilton, P. , & Gilliom, R. (2009). Quality of ground water from private domestic wells. Water Well Journal, April, 33–37.

[jeq270296-bib-0013] Diston, D. , Sinreich, M. , Zimmermann, S. , Baumgartner, A. , & Felleisen, R. (2015). Evaluation of molecular‐ and culture‐dependent MST markers to detect fecal contamination and indicate viral presence in good quality groundwater. Environmental Science & Technology, 49(12), 7142–7151. 10.1021/acs.est.5b00515 25871525

[jeq270296-bib-0014] Dubrovsky, N. M. , Burow, K. R. , Clark, G. M. , Gronberg, J. A. M. , Hamilton, P. A. , Hitt, K. J. , Mueller, D. K. , Munn, M. D. , Nolan, B. T. , Puckett, L. J. , Rupert, M. G. , Short, T. M. , Spahr, N. E. , & Wilber, W. G. (2010). The quality of our nation's water—nutrients in the nation's streams and groundwater, 1992–2004 (Circular 1350). U.S. Geological Survey.

[jeq270296-bib-0015] Erb, K. , Ronk, E. , Koundinya, V. , & Luczaj, J. (2015). Groundwater quality changes in a karst aquifer of northeastern Wisconsin, USA: reduction of brown water incidence and bacterial contamination resulting from implementation of regional task force recommendations. Resources, 4(3), 655–672. 10.3390/resources4030655

[jeq270296-bib-0016] Garson, G. D. (2013). Generalized linear models/generalized estimating equations. Statistical Associates Publishers.

[jeq270296-bib-0017] Green, C. T. , Fisher, L. H. , & Bekins, B. A. (2008). Nitrogen fluxes through unsaturated zones in five agricultural settings across the United States. Journal of Environmental Quality, 37(3), 1073–1085. 10.2134/jeq2007.0010 18453429

[jeq270296-bib-0018] Hamilton, S. K. (2012). Biogeochemical time lags may delay responses of streams to ecological restoration. Freshwater Biology, 57(S1), 43–57. 10.1111/j.1365-2427.2011.02685.x

[jeq270296-bib-0019] Hamlin, Q. F. , Martin, S. L. , Kendall, A. D. , & Hyndman, D. W. (2022). Examining relationships between groundwater nitrate concentrations in drinking water and landscape characteristics to understand health risks. GeoHealth, 6(5), e2021GH000524. 10.1029/2021GH000524 PMC9060635 35509496

[jeq270296-bib-0020] Hastie, T. , Friedman, J. , & Tibshirani, R. (2001). The elements of statistical learning: Data mining, inference, and prediction (1st ed.). Springer.

[jeq270296-bib-0021] Hunt, R. J. , & Johnson, W. P. (2017). Pathogen transport in groundwater systems: Contrasts with traditional solute transport. Hydrogeology Journal, 25(4), 921–930. 10.1007/s10040-016-1502-z

[jeq270296-bib-0022] Hynds, P. D. , Misstear, B. D. , & Gill, L. W. (2012). Development of a microbial contamination susceptibility model for private domestic groundwater sources. Water Resources Research, 48(12), 2012WR012492. 10.1029/2012WR012492

[jeq270296-bib-0023] Juckem, P. F. , Corson‐Dosch, N. T. , Schachter, L. A. , Green, C. T. , Ferin, K. M. , Booth, E. G. , Kucharik, C. J. , Austin, B. P. , & Kauffman, L. J. (2024). Design and calibration of a nitrate decision support tool for groundwater wells in Wisconsin, USA. Environmental Modelling & Software, 176, 105999. 10.1016/j.envsoft.2024.105999

[jeq270296-bib-0024] Kewaunee County Ordinances . (2016). Kewaunee County Agricultural Performance Standards . https://www.kewauneeco.org/i/f/files/Land%20%26%20Water%20Conservation/Chapter%2039.pdf

[jeq270296-bib-0025] Kewaunee County, WI . (2025). Land & water conservation . Kewaunee County, WI. https://www.kewauneeco.org/departments/land‐water‐conservation/

[jeq270296-bib-0026] Knobeloch, L. , Gorski, P. , Christenson, M. , & Anderson, H. (2013). Private drinking water quality in rural Wisconsin. Journal of Environmental Health, 75(7), 16–20. 23505770

[jeq270296-bib-0027] Kuehner, K. J. , Runkel, A. C. , & Barry, J. D. (2025). Informing nitrate concentration trends: Estimating groundwater residence time in a karstic, multiaquifer system using anthropogenic tracers (Minnesota, USA). Hydrogeology Journal, 33(1), 167–192. 10.1007/s10040-024-02871-2

[jeq270296-bib-0028] Lee, D. , & Murphy, H. M. (2020). Private wells and rural health: Groundwater contaminants of emerging concern. Current Environmental Health Reports, 7(2), 129–139. 10.1007/s40572-020-00267-4 31994010

[jeq270296-bib-0029] Liang, K.‐Y. , & Zeger, S. L. (1986). Longitudinal data analysis using generalized linear models. Biometrika, 73(1), 13–22. 10.1093/biomet/73.1.13

[jeq270296-bib-0030] Lin, L. , St Clair, S. , Gamble, G. D. , Crowther, C. A. , Dixon, L. , Bloomfield, F. H. , & Harding, J. E. (2023). Nitrate contamination in drinking water and adverse reproductive and birth outcomes: A systematic review and meta‐analysis. Scientific Reports, 13(1), 563. 10.1038/s41598-022-27345-x PMC9834225 36631499

[jeq270296-bib-0031] Luczaj, J. A. (2013). Geology of the Niagara escarpment in Wisconsin. Geoscience Wisconsin, 22, 1–34.

[jeq270296-bib-0032] Luczaj, J. A. , Konrad, A. , Norfleet, M. , & Schauer, A. (2023). Stable isotopic evaluation of recharge into a karst aquifer in a glaciated agricultural region of northeastern Wisconsin, USA. Hydrology, 10(6), 133. 10.3390/hydrology10060133

[jeq270296-bib-0033] Malecki, K. M. C. , Schultz, A. A. , Severtson, D. J. , Anderson, H. A. , & VanDerslice, J. A. (2017). Private‐well stewardship among a general population based sample of private well‐owners. Science of the Total Environment, 601–602, 1533–1543. 10.1016/j.scitotenv.2017.05.284 PMC5662198 28605871

[jeq270296-bib-0034] Masarik, K. (2023). Kewaunee County water wellness . https://www.kewauneeco.org/i/f/files/Land%20%26%20Water%20Conservation/Kewaunee%20County%20Oct%202023%20Well%20Testing%20Presentation.pdf

[jeq270296-bib-0035] McNutt, L.‐A. (2003). Estimating the relative risk in cohort studies and clinical trials of common outcomes. American Journal of Epidemiology, 157(10), 940–943. 10.1093/aje/kwg074 12746247

[jeq270296-bib-0036] Michigan State University . (2026). Michigan enviroimpact (Beta) . Michigan State University. https://enviroimpact.iwr.msu.edu/

[jeq270296-bib-0037] Minnesota Department of Agriculture . (2026). Runoff risk advisory forecast . Minnesota Department of Agriculture. https://www.mda.state.mn.us/protecting/cleanwaterfund/toolstechnology/runoffrisk

[jeq270296-bib-0038] Muldoon, M. A. , & Bradbury, K. R. (2010). Assessing seasonal variations in recharge and water quality in the Silurian aquifer in areas with thicker soil cover (Final Report Nos. 2019–04). Wisconsin Department of Natural Resources. https://wgnhs.wisc.edu/catalog/publication/000970/resource/wofr201904

[jeq270296-bib-0039] Muldoon, M. A. , Simo, J. T. , & Bradbury, K. (2001). Correlation of hydraulic conductivity with stratigraphy in a fractured‐dolomite aquifer, northeastern Wisconsin, USA. Hydrogeology Journal, 9(6), 570–583. 10.1007/s10040-001-0165-5

[jeq270296-bib-0040] Murphy, H. M. , McGinnis, S. , Blunt, R. , Stokdyk, J. , Wu, J. , Cagle, A. , Denno, D. M. , Spencer, S. , Firnstahl, A. , & Borchardt, M. A. (2020). Septic systems and rainfall influence human fecal marker and indicator organism occurrence in private wells in Southeastern Pennsylvania. Environmental Science & Technology, 54(6), 3159–3168. 10.1021/acs.est.9b05405 32073835

[jeq270296-bib-0041] Murray, A. , Hall, A. , Weaver, J. , & Kremer, F. (2021). Methods for estimating locations of housing units served by private domestic wells in the United States applied to 2010. Journal of the American Water Resources Association, 57(5), 828–843. 10.1111/1752-1688.12937 PMC8722366 34987281

[jeq270296-bib-0042] National Integrated Drought Information System (NIDIS) . (2025). U.S. drought monitor . NIDIS. https://www.drought.gov/historical‐information

[jeq270296-bib-0043] National Oceanic and Atmospheric Administration (NOAA) . (2019). NOAA runoff risk . NOAA. https://vlab.noaa.gov/web/noaa‐runoff‐risk

[jeq270296-bib-0044] Natural Resources Conservation Service (NRCS) . (2022). Web soil survey 3.4.0 . USDA, NRCS. https://websoilsurvey.nrcs.usda.gov/app/HomePage.htm

[jeq270296-bib-0045] Pan, W. (2001). Akaike's information criterion in generalized estimating equations. Biometrics, 57(1), 120–125. 10.1111/j.0006-341X.2001.00120.x 11252586

[jeq270296-bib-0046] Petculescu, I. , Hynds, P. , Brown, R. S. , McDermott, K. , & Majury, A. (2022). An assessment of total coliforms and associated thresholds as water quality indicators using a large Ontario private drinking water well dataset. Science of the Total Environment, 846, 157478. 10.1016/j.scitotenv.2022.157478 35868388

[jeq270296-bib-0047] Ransom, K. M. , Nolan, B. T. , Stackelberg, P. E. , Belitz, K. , & Fram, M. S. (2022). Machine learning predictions of nitrate in groundwater used for drinking supply in the conterminous United States. Science of the Total Environment, 807, 151065. 10.1016/j.scitotenv.2021.151065 34673076

[jeq270296-bib-0048] Stokdyk, J. , Firnstahl, A. , Bradbury, K. , Muldoon, M. , Kieke, B. , & Borchardt, M. A. (2025). Sources and risk factors for nitrate, pathogens, and fecal contamination of private wells in rural southwestern Wisconsin, USA. Water Research, 275, 123202. 10.1016/j.watres.2025.123202 39892189

[jeq270296-bib-0049] U.S. Department of Agriculture National Agricultural Statistics Service (USDA NASS) . (2024). 2022 Census of agriculture; Wisconsin state and County data (AC‐22‐A‐49). USDA NASS. https://www.nass.usda.gov/Publications/AgCensus/2022/Full_Report/Volume_1,_Chapter_2_County_Level/Wisconsin/

[jeq270296-bib-0050] U.S. Geological Survey (USGS) . (2025). Water data for the nation . USGS. 10.5066/F7P55KJN

[jeq270296-bib-0051] Van Meter, K. J. , Basu, N. B. , Veenstra, J. J. , & Burras, C. L. (2016). The nitrogen legacy: Emerging evidence of nitrogen accumulation in anthropogenic landscapes. Environmental Research Letters, 11(3), 035014. 10.1088/1748-9326/11/3/035014

[jeq270296-bib-0052] Voisin, J. , Cournoyer, B. , Vienney, A. , & Mermillod‐Blondin, F. (2018). Aquifer recharge with stormwater runoff in urban areas: Influence of vadose zone thickness on nutrient and bacterial transfers from the surface of infiltration basins to groundwater. Science of the Total Environment, 637–638, 1496–1507. 10.1016/j.scitotenv.2018.05.094 29801243

[jeq270296-bib-0053] Wisconsin Administrative Code, NR 151.075 Silurian bedrock performance standards . (2018). https://docs.legis.wisconsin.gov/code/admin_code/nr/100/151/ii/075

[jeq270296-bib-0054] Wisconsin Department of Agriculture, Trade and Consumer Protection (DATCP) . (2024). Wisconsin groundwater quality: Agricultural chemicals in Wisconsin groundwater . DATCP. https://datcp.wi.gov/Documents2/2023StatewideGroundwaterSurveyReport.pdf

[jeq270296-bib-0055] Wisconsin Manure Management Advisory System . (2025). Runoff risk advisory forecast . Wisconsin Manure Management Advisory System. https://runoffriskadvisory.wi.gov/

[jeq270296-bib-0056] Worthington, S. R. H. , & Smart, C. C. (2017). Transient bacterial contamination of the dual‐porosity aquifer at Walkerton, Ontario, Canada. Hydrogeology Journal, 25(4), 1003–1016. 10.1007/s10040-016-1514-8

