# Supplementary material for: Association of runoff risk with private well contamination in a fractured dolostone aquifer
Source: J Environ Qual. 2026 Oct 29;55(6):e70296. doi: 10.1002/jeq2.70296 (PMC13717291; doi:10.1002/jeq2.70296)
Supplement: Supplementary file 1 [file JEQ2-55-0-s001.docx]

**Supplemental Material for:**

Association of modeled nutrient runoff risk with private well contamination in a fractured dolostone aquifer

Joe Heffron^1,2^*, Joel P. Stokdyk^2,3^, Tucker R. Burch^1,2^, Rachel M. Cook^1,2^, Aaron D. Firnstahl^2,3^, Eric O. Young^1^, Jessica F. Sherman^1^, Dustin Goering^4^, Burney A. Kieke Jr.^5^, Mark A. Borchardt^1,2^

Affiliations:

^1^ U.S. Department of Agriculture-Agricultural Research Service, Environmentally Integrated Dairy Management Research Unit, Marshfield, WI, United States

^2^ U.S. Geological Survey and U.S. Department of Agriculture-Agricultural Research Service, Laboratory for Infectious Disease and the Environment, Marshfield, WI, United States

^3^ U.S. Geological Survey, Upper Midwest Water Science Center, Marshfield, WI, United States

^4^ U.S. National Weather Service, North Central River Forecast Center, Chanhassen, Minnesota, USA

^5^ Center for Clinical Epidemiology and Population Health, Marshfield Clinic Research Institute, Marshfield, Wisconsin, United States

* Corresponding Author: [joseph.heffron@usda.gov](mailto:joseph.heffron@usda.gov)

Table of contents

[Table of Contents	2](#_Toc241056225)

[List of Tables	2](#_Toc241056226)

[List of Figures	2](#_Toc241056227)

[S1.	Environmental microbiology methods and controls	3](#_Toc241056228)

[S1.1.	Detailed total coliforms method	3](#_Toc241056229)

[S1.2.	Detailed qPCR methods	3](#_Toc241056230)

[S1.3.	Quality control measures	6](#_Toc241056231)

[S2.	Selection of days-prior windows for delay in contamination	8](#_Toc241056232)

[S3.	Summary of contamination outcomes	10](#_Toc241056233)

[S4.	Comparison of runoff risk using observed vs predicted weather data	13](#_Toc241056234)

[S5.	Association of nitrate contamination with well siting and construction	14](#_Toc241056235)

List of Tables

[Table S1. Primer and probe sequences and concentrations for qPCR.	4](#_Toc241056084)

[Table S2. Standard curve metrics for targets analyzed.	5](#_Toc241056085)

[Table S3. Comparison of nitrate concentration models using Quasilikelihood under the Independence model Criterion (QICu).	9](#_Toc241056086)

[Table S4. Summary of outcomes by sampling date.	10](#_Toc241056087)

[Table S5. Summary of outcomes by well.	11](#_Toc241056088)

[Table S6. Depth to bedrock categories for the wells sampled in this study.	15](#_Toc241056089)

List of Figures

[Figure S1. Environmental Microbiology Minimum Information (EMMI) checklist	7](#_Toc241056064)

[Figure S2. Comparison of runoff risk computed using observed vs. predicted weather conditions	13](#_Toc241056065)

[Figure S3. Characteristics of wells that never, sometimes, or always exceeded national background levels of nitrate (1 NO_3_-N L^-1^) during the study	14](#_Toc241056066)

# S1. Environmental microbiology methods and controls

## S1.1. Detailed total coliforms method

Total coliforms were analyzed using the IDEXX Colilert kit (Westbrook, ME). A 100 mL subsample of each 500 mL grab sample was measured out into pre-sterilized bottles, mixed with the kit reagents, poured into Quanti-trays (IDEXX), and sealed. Trays were incubated at 35° C for 24-28 hours before counting. One negative control (100 mL of DI water) and one positive control (IDEXX-QC Coliform and *E. coli* Kit) were processed per sampling event.

## S1.2. Detailed qPCR methods

For manual lysis, Buffer AVL was added directly onto the filter and tubes were mixed by vortexing and incubated at room temperature for 10 minutes (560 µL Buffer AVL was used for samples from 11/16/2022; 700 µL was used for the rest of the samples). Tubes were centrifuged at 500×g for 15 seconds, and 560 µL of lysed sample was added to a 2 mL tube and transferred to the QIAcube Connect for automated column-based extraction. 140 µL was eluted off the columns after extraction. The eluted template was stored at 4°C until used for analysis.

For qPCR, the total reaction volume of 20 µL included 14 µL of master mix and 6 µL of extracted sample. The thermal cycling program for all assays started with 95°C for 5 minutes followed by 45 cycles of 10 seconds at 95°C and 1 minute at 60°C with ramp rates of 4.4 and 2.2°C per second. Primers and probes are detailed in Table S1.

Standard curves were serial dilutions of positive controls in AE buffer with 0.02% bovine serum albumin; concentrations ranged from 1 to 10^6^ gene copies (gc)/reaction. Cq values were calculated by second derivative maximum and regressed against log_10_ marker concentrations using the LightCycler® 480 software’s nonlinear function (Roche Diagnostics, Penzburg, Germany). Standard curve parameters and 95% limits of detection are reported in Table S2.

Table S1. Primer and probe sequences and concentrations for qPCR.

| Microorganism (gene marker) | Primer/  probe | Sequence 5' to 3' | Primer/probe conc. (nM) | Amplicon size (bp) | Reference |
| --- | --- | --- | --- | --- | --- |
| Bacteroidales-like CowM2 (DHIG domain protein) | Forward | CGGCCAAATACTCCTGATCGT | 400 | 92 | (Shanks et al., 2008) |
|  | Reverse | GCTTGTTGCGTTCCTTGAGATAAT | 400 |  |  |
|  | Probe | AGGCACCTATGTCCTTTACCTCATCAACTACAGACA | 50 |  |  |
| Bacteroidales-like CowM3 (HD super family hydrolase) | Forward | CCTCTAATGGAAAATGGATGGTATCT | 500 | 122 | (Shanks et al., 2008) |
|  | Reverse | CCATACTTCGCCTGCTAATACCTT | 500 |  |  |
|  | Probe | TTATGCATTGAGCATCGAGGCC | 50 |  |  |
| Bovine adenovirus (serotypes 4-8; hexon) | Forward | CRAGGGAATAYYTGTCTGAAAATC | 500 | 87 | (Wong & Xagoraraki, 2010) |
|  | Reverse | AAGGATCTCTAAATTTYTCTCCAAGA | 500 |  |  |
|  | Probe | TTCATCWCTGCCACWCAAAGCTTTTTT | 50 |  |  |
| Bovine herpes virus (positive DNA extraction control; beta-actin) | Forward | GCAATGCTGCAGGACTAGGTATAAT | 500 | 97 | (Wang et al., 2007) |
|  | Reverse | ACACTGTAATTGATGACCCCATTCT | 500 |  |  |
|  | Probe | ACCAAGACTTGTATGATGCTGCCAAAGCA | 50 |  |  |
| Bovine polyomavirus (*VP1*) | Forward | TGGCTTTCTGACTCAGCCAAA | 500 | 79 | (Wong & Xagoraraki, 2011) |
|  | Reverse | TCTCTTCCTGAGAGTCACAGACATG | 500 |  |  |
|  | Probe | ACCAACAGCAATTTAGAGGCCTTCCCAG | 50 |  |  |
| *Moraxella bovis* (process control; 16s rRNA) | Forward | GGTGACGACCGCTTGTTT | 500 | 63 | (Zheng et al., 2019) |
|  | Reverse | ATCATCGCCTTCATCTCCAG | 500 |  |  |
|  | Probe | CGATCGTTGCCTTTACCACC | 50 |  |  |
| Ruminant *Bacteroides* (16SrRNA) (Rum-2-Bac) | Forward | ACAGCCCGCGATTGATACTGGTAA | 500 | 99 | (Mieszkin et al., 2010) |
|  | Reverse | CAATCGGAGTTCTTCGTGAT | 500 |  |  |
|  | Probe | ATGAGGTGGATGGAATTCGTGGTGT + MGB | 50 |  |  |
| Sketa 22 (PCR inhibition control; rRNA of chum salmon) | Forward | GGTTTCCGCAGCTGGG | 500 | 77 | (Haugland et al., 2005) |
|  | Reverse | CCGAGCCGTCCTGGTCTA | 500 |  |  |
|  | Probe | AGTCGCAGGCGGCCACCGT | 50 |  |  |

Table S2. Standard curve metrics for targets analyzed.

| Organism and/or (assay name) | Curve ID | Error | R^2^  (1-Error) | Efficiency | Slope | y-intercept | Max  Cq | Standard containing Max Cq | Lowest Standard | Assay 95% LOD (gene copies/reaction) |
| --- | --- | --- | --- | --- | --- | --- | --- | --- | --- | --- |
| Bovine adenovirus (BAV4-8) | 1 | 0.033 | 0.967 | 1.962 | -3.416 | 39.73 | 39.22 | 10 | 10 | 5.3 |
|  | 2 | 0.0497 | 0.9503 | 1.995 | -3.334 | 39.43 | 40 | 10 | 10 |  |
| Bovine polyomavirus (BPyV) | 1 | 0.0154 | 0.9846 | 1.864 | -3.698 | 43.03 | 40 | 9, 10 | 10 | 2.3 |
|  | 2 | 0.0018 | 0.9982 | 1.919 | -3.533 | 41.87 | 40 | 10 | 10 |  |
| Bacteroidales-like (CowM2) | 1 | 0.0498 | 0.9502 | 1.953 | -3.441 | 41.54 | 40 | 10 | 10 | 4.5 |
|  | 2 | 0.0189 | 0.9811 | 1.95 | -3.448 | 41.59 | 38.92 | 9 | 10 |  |
| Bacteroidales-like (CowM3) | 1 | 0.0107 | 0.9893 | 1.959 | -3.425 | 39.8 | 37.88 | 10 | 10 | 11.9 |
|  | 2 | 0.00427 | 0.99573 | 1.957 | -3.429 | 40.57 | 40 | 10 | 10 |  |
| Ruminant *Bacteroides* (Rum-2-Bac) | 1 | 0.00925 | 0.99075 | 1.948 | -3.453 | 41.97 | 40 | 9, 10 | 10 | 6.1 |
|  | 2 | 0.00902 | 0.99098 | 1.978 | -3.375 | 41.68 | 40 | 10 | 10 |  |

## S1.3. Quality control measures

Controls were established throughout sample processing and analysis, as summarized in the Environmental Microbiology Minimum Information (EMMI) checklist [Figure S1; (Borchardt, Boehm, et al., 2021)].

**Sample concentration.** Two negative controls (1 L of RO water with *M. bovis* and MgCl_2_ added) were filtered per sampling event, one before and one after filtration of samples. All filter negative controls were negative for all targets analyzed.

**Extraction.** One positive extraction control was run per extraction batch using bovine herpes virus vaccine (Zoetis Inc., Parsippany, NJ) in place of the sample filter. Two extraction negative controls were run per batch using AE buffer. All extraction negative controls were negative for all targets, except for one that was positive for Bacteroidales-like CowM2. No samples were positive for Bacteroidales-like CowM2 in the affected batch.

**qPCR.** Inhibition in the qPCR step was tested for all samples by adding Salmon testes DNA (Sketa-22; IDT) to all samples as well as an inhibition control, containing 6 µL of AE buffer with 0.02% BSA. Samples with Cq values two or more cycles higher than the inhibition control were considered inhibited. Six samples from one well showed inhibition and were diluted 1:5 or 1:10 with AE buffer (Qiagen).

Negative and positive controls were run in duplicate for each target and LightCycler® 480 II run. Negative controls used 6 µL of AE buffer in place of extracted sample while positive controls used 6 µL of the target Ultramer sequences (IDT). All qPCR negative controls were negative for all targets, and all qPCR positive controls for all targets were within 0.5 cycles of expected values.

Figure S1. Environmental Microbiology Minimum Information (EMMI) checklist

# S2. Selection of days-prior windows for delay in contamination

To account for delays in contaminant transport to groundwater, time windows for relevant runoff risk prior to the anchor (sampling) date were identified using a screening process. Maximum Runoff Risk Advisory Forecast (RRAF) percent was evaluated in three-day windows from days 1-21.

Total coliform detections had the greatest strength of association with runoff risk for days 4-6 (*p* = 0.0060, 2-sided Fisher’s exact test), 5-7 (*p* = 0.0060), and 6-8 (*p* = 0.0014). The results were identical for the day 4-6 and day 5-7 windows, indicating that the maximum runoff risk values for each sample occurred on days that overlap the two windows (i.e., days 5 and 6 prior to sampling). For the 4-6 and 5-7 windows, the detection rate ratios for Low, Moderate and Severe versus “No runoff event expected” were 1.69, 3.72 and 3.38 respectively (the latter ratio was based on a total of 12 samples in the Severe category which makes it more difficult to interpret due to random variation) with the denominator detection rate for the “No runoff event expected” category being 7.41%. The day 6-8 window, which had a slightly more significant association with total coliforms detection, had corresponding rate ratios of 3.36, 3.92, and 3.58 with the denominator detection rate being 7.45%. However, the pattern in these ratios was more difficult to interpret since there were substantially fewer samples in the Low/Moderate/Severe categories (combined n=47 versus n=73 for the 4-6 and 5-7 windows). The Low category for the 6-8 window had a much smaller sample size (n=8) compared to the 4-6 and 5-7 windows (n=32). Based on the sample size considerations noted above for the day 6-8 window and because the day 5-7 window was intermediate between days 4-6 and 6-8, the day 5-7 window was selected for further analysis of total coliforms.

For bovine MST, detections in the day 1-3 window had a moderate association with categorical runoff risk (*p* = 0.1178, 2-sided Fisher’s exact test). The only windows with significant associations at an alpha value of 0.05 occurred much later (days 12-14, *p* = 0.0095; days 13-15, *p* = 0.0270). The observed detection rate ratio patterns across runoff risk categories for the day 1-3 window (no elevated detection for Low and twofold elevation for Moderate; no samples fell into the Severe category for this window) seemed more plausible than those for the day 12-14 and 13-15 windows (fluctuating detection from Low to Severe with a spike at Low). Previous research in the same and similar hydrologic settings had found rapid transport of fecal indicator microbes to private wells within one week (Arnaud et al., 2015; Borchardt, Stokdyk, et al., 2021). Furthermore, we found the later time windows improbable based on the lack of association between bovine MST detections and any intermediate window between 1-3 days and 12-14 days, as well as the significant association of total coliform detections within one week. Lastly, our goal was to give homeowners an early warning system, so the earliest possible breakthrough of bovine fecal contamination was considered most critical. Therefore, we selected the day 1-3 window for further analysis.

For nitrate concentration, the initial step in the assessment of time windows was to generate and examine, for each candidate 3-day window (1-3, 2-4,…,19-21), scatterplots with the natural log of nitrate concentration on the vertical axis and maximum runoff risk (from zero to the maximum observed value in increments of 5%) on the horizontal axis. Nitrate concentration was natural log transformed to emulate subsequent gamma regression models. Two scatterplot smoothers (loess and penalized B-splines) were overlaid on the plots. A pattern observed for several windows was segment(s) of data sparsity across the observed maximum runoff risk range. Due to this phenomenon, and because elevated nitrate concentrations were expected to be delayed relative to microbial detections and persist longer due to soil organic nitrogen reserves (Section 4.1), detailed model-based assessment was restricted to windows 9-11 through 16-18. This entailed fitting, for each window being assessed, gamma regression models with a natural log link function, nitrate concentration as the dependent variable, and maximum runoff risk (from zero to the maximum observed value in increments of 5%) as the independent variable. Maximum runoff risk percent was represented as a natural cubic spline in all regression models. For each window, separate models were fit with spline knots at the quartiles and quintiles of maximum runoff risk percent. The primary results derived from the models were 1) Quasilikelihood under the Independence model Criterion (QICu) goodness-of-fit statistics, and 2) graphics depicting estimated ratios of mean nitrate concentrations for each observed maximum runoff risk percent value > 0 versus when maximum runoff risk percent = 0 (along with 95% pointwise confidence bands). The four best-fitting models (lowest QICu) used the day 9-11 and 10-12 windows and had QICu values ranging from 143.41 to 146.91 while the corresponding range for the remaining models was 153.40-176.12 (Table S3). In the absence of a scientific rationale for selecting among these models, and since each of them had similar ratio graphics, we chose the one with the lowest QICu (i.e., the model based on the day 10-12 window with spline knots at maximum runoff risk percent quartiles). To establish whether any of the windows that were less rigorously evaluated (i.e., those outside of the 9-11 through 16-18 range) achieved better model fit than the selected window, corresponding QICu statistics were computed. The QICu range for this group was 156.35-200.22.

Table S3. Comparison of nitrate concentration models using Quasilikelihood under the Independence model Criterion (QICu). The model for quintiles/15-17 did not converge.

| Spline knots | Days prior range | QICu |
| --- | --- | --- |
| quartiles | 10-12 | 143.41 |
| quartiles | 9-11 | 143.61 |
| quintiles | 9-11 | 146.12 |
| quintiles | 10-12 | 146.91 |
| quartiles | 15-17 | 153.40 |
| quintiles | 16-18 | 165.37 |
| quintiles | 11-13 | 165.98 |
| quartiles | 11-13 | 167.44 |
| quartiles | 14-16 | 168.41 |
| quartiles | 16-18 | 168.54 |
| quintiles | 12-14 | 168.76 |
| quintiles | 14-16 | 172.99 |
| quintiles | 13-15 | 173.69 |
| quartiles | 13-15 | 174.20 |
| quartiles | 12-14 | 176.12 |

# S3. Summary of contamination outcomes

Table S4. Summary of outcomes by sampling date.

|  |  | Nitrate | | | Total coliforms | | Bovine MST | |
| --- | --- | --- | --- | --- | --- | --- | --- | --- |
| Date | Wells | Mean concentration | Exceedance* | Above background^†^ | Detect | Nondetect | Detect | Nondetect |
|  | *n* | mg-N L^-1^ | *n* | *N* | *n* | *n* | *n* | *n* |
| 16-Nov-22 | 33 | - Not measured - | | | 2 | 31 | 3 | 30 |
| 8-Feb-23 | 33 | 2.10 | 2 | 10 | 1 | 32 | 8 | 25 |
| 19-Apr-23 | 34 | 2.87 | 4 | 12 | 6 | 28 | 4 | 30 |
| 26-Apr-23 | 33 | 2.76 | 4 | 11 | 5 | 28 | 5 | 28 |
| 11-Jul-23 | 36 | 2.38 | 4 | 13 | 2 | 34 | 3 | 33 |
| 1-Nov-23 | 34 | 1.82 | 1 | 12 | 8 | 26 | 2 | 32 |
| 19-Dec-23 | 32 | 2.02 | 2 | 11 | 3 | 29 | 6 | 26 |

* MCL: 10 mg NO_3_-N L^-1^

^†^ National background level: 1 mg NO_3_-N L^-1^ (Dubrovsky et al., 2010)

Table S5. Summary of outcomes by well.

|  | Nitrate | | | | Total coliforms | | Bovine MST | |
| --- | --- | --- | --- | --- | --- | --- | --- | --- |
| Well ID | Mean concentration | Exceedance* | Above background^†^ | Dates sampled | Detect | Nondetect | Detect | Nondetect |
|  | mg-N L^-1^ | *n* | *n* | *n* | *n* | *n* | *n* | *n* |
| KW001 | <0.002 | 0 | 0 | 5 | 1 | 5 | 1 | 5 |
| KW019 | 3.73 | 0 | 6 | 6 | 0 | 7 | 5 | 2 |
| KW031 | <0.002 | 0 | 0 | 6 | 0 | 7 | 0 | 7 |
| KW032 | <0.002 | 0 | 0 | 6 | 2 | 5 | 1 | 6 |
| KW041 | 8.32 | 3 | 6 | 6 | 1 | 6 | 0 | 7 |
| KW046 | <0.002 | 0 | 0 | 6 | 0 | 7 | 2 | 5 |
| KW049 | 2.34 | 0 | 3 | 5 | 0 | 5 | 1 | 4 |
| KW054 | <0.002 | 0 | 0 | 4 | 0 | 5 | 0 | 5 |
| KW057 | 0.690 | 0 | 2 | 6 | 0 | 7 | 0 | 7 |
| KW061 | <0.002 | 0 | 0 | 1 | 0 | 1 | 1 | 0 |
| KW070 | 0.074 | 0 | 0 | 6 | 0 | 7 | 2 | 5 |
| KW073 | 12.6 | 4 | 6 | 6 | 0 | 7 | 0 | 7 |
| KW078 | 0.087 | 0 | 0 | 6 | 2 | 5 | 1 | 6 |
| KW080 | <0.002 | 0 | 0 | 6 | 0 | 7 | 0 | 7 |
| KW087 | 0.103 | 0 | 0 | 5 | 0 | 6 | 1 | 5 |
| KW093 | <0.002 | 0 | 0 | 4 | 0 | 5 | 0 | 5 |
| KW094 | <0.002 | 0 | 0 | 5 | 0 | 6 | 1 | 5 |
| KW101 | <0.002 | 0 | 0 | 6 | 0 | 7 | 0 | 7 |
| KW105 | 14.0 | 6 | 6 | 6 | 6 | 1 | 1 | 6 |
| KW110 | <0.002 | 0 | 0 | 6 | 0 | 7 | 1 | 6 |
| KW111 | 0.189 | 0 | 0 | 6 | 0 | 7 | 1 | 6 |
| KW113 | 0.050 | 0 | 0 | 6 | 0 | 7 | 3 | 4 |
| KW123 | <0.002 | 0 | 0 | 6 | 0 | 7 | 0 | 7 |
| KW131 | 7.71 | 2 | 6 | 6 | 3 | 3 | 1 | 5 |

Table S5 continued. Summary of outcomes by well.

|  | Nitrate | | | | Total coliforms | | Bovine MST | |
| --- | --- | --- | --- | --- | --- | --- | --- | --- |
| Well ID | Mean concentration | Exceedance* | Above background^†^ | Dates sampled | Detect | Nondetect | Detect | Nondetect |
|  | mg-N L^-1^ | *n* | *n* | *n* | *n* | *n* | *n* | *n* |
| KW132 | 4.16 | 0 | 6 | 6 | 0 | 7 | 1 | 6 |
| KW135 | 0.059 | 0 | 0 | 6 | 0 | 7 | 2 | 5 |
| KW143 | <0.002 | 0 | 0 | 6 | 1 | 6 | 1 | 6 |
| KW146 | 9.52 | 2 | 6 | 6 | 2 | 5 | 1 | 6 |
| KW153 | <0.002 | 0 | 0 | 6 | 0 | 7 | 1 | 6 |
| KW170 | 1.64 | 0 | 6 | 6 | 0 | 7 | 1 | 6 |
| KW178 | 4.46 | 0 | 6 | 6 | 0 | 7 | 0 | 7 |
| KW185 | 3.39 | 0 | 4 | 5 | 2 | 4 | 0 | 6 |
| KW189 | <0.002 | 0 | 0 | 6 | 0 | 7 | 0 | 7 |
| KW190 | 0.075 | 0 | 0 | 6 | 0 | 7 | 1 | 6 |
| KW191 | 5.79 | 0 | 6 | 6 | 4 | 3 | 0 | 7 |
| KW199 | <0.002 | 0 | 0 | 6 | 3 | 4 | 0 | 7 |

* MCL: 10 mg NO_3_-N L^-1^

^†^ National background level: 1 mg NO_3_-N L^-1^ (Dubrovsky et al., 2010)

# S4. Comparison of runoff risk using observed vs predicted weather data

This study established a connection between modeled runoff risk and groundwater contamination using RRAF outputs based on observed weather. To assess the feasibility of using RRAF outputs based on predicted weather, we compared our observed weather outputs to predicted outputs from the Wisconsin RRAF online tool (<https://runoffriskadvisory.wi.gov/>) over 14 months spanning the study period (Oct. 28, 2022 – Dec. 31, 2023). For the predicted data, we used 24-hour RRAF predictions generated daily at approximately 4 am. Observations during winter conditions were excluded, because predicted output from the web RRAF tool included only categorical risk values for winter conditions. RRAF predictions were highly correlated to observed outputs (Pearson’s product-moment correlation = 0.850 ± 0.005,
*p* < 2.2x10^-16^; Figure S2). In this study, 75% runoff risk was identified as a threshold above which contamination was more likely compared to a baseline of 0% runoff risk. Weather predictions correctly identified runoff risk above this threshold in 70.8% of cases (Recall = 438/619) and below this threshold in 98.5% of cases (Specificity = 10458/10612). Thus, we expect weather predictions to introduce additional uncertainty to the association between runoff risk and contamination.

Figure S2. Comparison of runoff risk computed using observed vs. predicted weather conditions. Individual points represent daily comparisons of observed and predicted risk for a given study well; points are represented at 5% opacity to emphasize overlapping data. Horizontal and vertical dashed lines (blue) denote risk values of 75%, above which contamination exceeded the baseline for nitrate, fecal coliforms, and bovine fecal markers.

# S5. Association of nitrate contamination with well siting and construction

In addition to runoff risk, between-well variability in exposure to manure sources, hydrogeology, and well construction may have influenced the contamination outcomes assessed in this study. To explore contributing factors to nitrate contamination, wells were categorized as testing “Always,” “Sometimes,” or “Never” above national background levels of nitrate [1 mg NO_3_-N L^-1^, (Dubrovsky et al., 2010)] throughout the study. This exploratory data analysis was limited to risk factors determined to be significant by Borchardt et al. (Borchardt, Stokdyk, et al., 2021). For nitrate, these risk factors were depth to bedrock, area of cultivated fields with a nutrient management plan (NMP) within 229 m, well casing depth, and length of well casing below the water table. Median values decreased monotonically for depth to bedrock and well casing depth (Figure S3). These results support the hypothesis that shallow wells, especially those occurring in areas with shallow depth to bedrock, are more susceptible to contamination from the surface and vadose zone. We expected to see a trend of greater nitrate contamination from wells situated near areas of intensive agriculture, but median values for area of fields with an NMP was similar for wells “Never,” “Sometimes,” and “Always” above background levels.

Figure S3. Characteristics of wells that never, sometimes, or always exceeded national background levels of nitrate (1 NO_3_-N L^-1^) during the study. Superscript values (red) are number of study wells in each category; well construction data were not available for some study wells. Boxes represent interquartile range (IQR) of the parameter (in meters except hectares for nutrient management plan (NMP) field area); whiskers represent minimum and maximum values up to 1.5 times the IQR.

Table S6. Depth to bedrock categories for the wells sampled in this study.

| Depth to Bedrock | Number of wells |
| --- | --- |
| m | *n* |
| < 1.5 | 2 |
| 1.5 - 6.1 | 15 |
| > 6.1 | 19 |
| Total | 36 |

REFERENCES

Arnaud, E., Best, A., Parker, B. L., Aravena, R., & Dunfield, K. (2015). Transport of *Escherichia coli* through a Thick Vadose Zone. *Journal of Environmental Quality*, *44*(5), 1424–1434. https://doi.org/10.2134/jeq2015.02.0067

Borchardt, M. A., Boehm, A. B., Salit, M., Spencer, S. K., Wigginton, K. R., & Noble, R. T. (2021). The Environmental Microbiology Minimum Information (EMMI) Guidelines: qPCR and dPCR Quality and Reporting for Environmental Microbiology. *Environmental Science & Technology*, *55*(15), 10210–10223. https://doi.org/10.1021/acs.est.1c01767

Borchardt, M. A., Stokdyk, J. P., Kieke, B. A., Muldoon, M. A., Spencer, S. K., Firnstahl, A. D., Bonness, D. E., Hunt, R. J., & Burch, T. R. (2021). Sources and Risk Factors for Nitrate and Microbial Contamination of Private Household Wells in the Fractured Dolomite Aquifer of Northeastern Wisconsin. *Environmental Health Perspectives*, *129*(6), 067004. https://doi.org/10.1289/EHP7813

Dubrovsky, N. M., Burow, K. R., Clark, G. M., Gronberg, J. A. M., Hamilton, P. A., Hitt, K. J., Mueller, D. K., Munn, M. D., Nolan, B. T., Puckett, L. J., Rupert, M. G., Short, T. M., Spahr, N. E., & Wilber, W. G. (2010). *The Quality of Our Nation’s Water—Nutrients in the Nation’s Streams and Groundwater, 1992–2004* (Circular 1350; p. 175). U.S. Geological Survey.

Haugland, R. A., Siefring, S. C., Wymer, L. J., Brenner, K. P., & Dufour, A. P. (2005). Comparison of Enterococcus measurements in freshwater at two recreational beaches by quantitative polymerase chain reaction and membrane filter culture analysis. *Water Research*, *39*(4), 559–568. https://doi.org/10.1016/j.watres.2004.11.011

Mieszkin, S., Yala, J. ‐F., Joubrel, R., & Gourmelon, M. (2010). Phylogenetic analysis of *Bacteroidales* 16S rRNA gene sequences from human and animal effluents and assessment of ruminant faecal pollution by real‐time PCR. *Journal of Applied Microbiology*, *108*(3), 974–984. https://doi.org/10.1111/j.1365-2672.2009.04499.x

Shanks, O. C., Atikovic, E., Blackwood, A. D., Lu, J., Noble, R. T., Domingo, J. S., Seifring, S., Sivaganesan, M., & Haugland, R. A. (2008). Quantitative PCR for Detection and Enumeration of Genetic Markers of Bovine Fecal Pollution. *Applied and Environmental Microbiology*, *74*(3), 745–752. https://doi.org/10.1128/AEM.01843-07

Wang, J., O’Keefe, J., Orr, D., Loth, L., Banks, M., Wakeley, P., West, D., Card, R., Ibata, G., Van Maanen, K., Thoren, P., Isaksson, M., & Kerkhofs, P. (2007). Validation of a real-time PCR assay for the detection of bovine herpesvirus 1 in bovine semen. *Journal of Virological Methods*, *144*(1–2), 103–108. https://doi.org/10.1016/j.jviromet.2007.04.002

Wisconsin Manure Management Advisory System. (2025). *Runoff Risk Advisory Forecast*. https://runoffriskadvisory.wi.gov/; Accessed: 11/25/2025.

Wong, K., & Xagoraraki, I. (2010). Quantitative PCR assays to survey the bovine adenovirus levels in environmental samples. *Journal of Applied Microbiology*, *109*(2), 605–612. https://doi.org/10.1111/j.1365-2672.2010.04684.x

Wong, K., & Xagoraraki, I. (2011). Evaluating the prevalence and genetic diversity of adenovirus and polyomavirus in bovine waste for microbial source tracking. *Applied Microbiology and Biotechnology*, *90*(4), 1521–1526. https://doi.org/10.1007/s00253-011-3156-z

Zheng, W., Porter, E., Noll, L., Stoy, C., Lu, N., Wang, Y., Liu, X., Purvis, T., Peddireddi, L., Lubbers, B., Hanzlicek, G., Henningson, J., Liu, Z., & Bai, J. (2019). A multiplex real-time PCR assay for the detection and differentiation of five bovine pinkeye pathogens. *Journal of Microbiological Methods*, *160*, 87–92. https://doi.org/10.1016/j.mimet.2019.03.024

Any use of trade, firm, or product names is for descriptive purposes only and does not imply endorsement by the U.S. Government.
